# Effects of 3 months of detraining following cardiac rehabilitation in patients with atrial fibrillation

**DOI:** 10.1186/s11556-022-00293-1

**Published:** 2022-04-29

**Authors:** Maria Borland, Lennart Bergfeldt, Åsa Cider, Agneta Rosenkvist, Marika Jakobsson, Kristin Olsson, Adam Lundwall, Lars Andersson, Lena Nordeman

**Affiliations:** 1 Närhälsan Sörhaga Rehabilitation Center, Alingsås, Sweden; 2 Region Västra Götaland, Research and Development Primary Health Care, Research and Development Center Södra Älvsborg, Borås Västra Götaland, Sweden; 3 Department of Health and Rehabilitation/Physiotherapy, Sahlgrenska Academy, Institute of Neuroscience and Physiology, University of Gothenburg, Gothenburg, Sweden; 4 SV Hospital Group rehabilitation Center, Alingsås Hospital, 441 83 Alingsås, Sweden; 5 Department of Molecular and Clinical Medicine, Sahlgrenska Academy, Institute of Medicine, University of Gothenburg, Gothenburg, Sweden; 6 Department of Cardiology, Sahlgrenska University Hospital, Gothenburg, Sweden; 7 Department of Occupational Therapy and Physiotherapy, Sahlgrenska University Hospital, Gothenburg, Sweden; 8 Habo Health Center, Bra Liv Health Center, Habo, Jönköping Sweden; 9 SV Hospital Group, Alingsås Hospital, Alingsås, Sweden

**Keywords:** Atrial fibrillation, Health- related quality of life, Physical fitness, Physical activity

## Abstract

**Background:**

Atrial fibrillation negatively impacts physical fitness and health-related quality of life. We recently showed that 3 months of physiotherapist-led exercise-based cardiac rehabilitation improves physical fitness and muscle function in elderly patients with permanent atrial fibrillation and concomitant diseases. Little is, however, known about the consequences for physical fitness, physical activity level, and health-related quality of life after ending the rehabilitation period.

**Methods:**

Prospective 3 months follow-up study of 38 patients out of 40 eligible (10 women) who, as part of a randomized controlled trial, had completed a 3 months physiotherapist-led cardiac rehabilitation resulting in improved physical fitness,. In the current study, the participants were instructed to refrain from exercise for 3 months after completion of the rehabilitation period. Primary outcome measure was physical fitness measured as highest achieved workload using an exercise tolerance test. Secondary outcome measures were muscle function (muscle endurance tests), physical activity level (questionnaire and accelerometer), and health-related quality of life, (Short Form-36), as in the preceding intervention study. We used the Wilcoxon Signed Rank test to analyse differences between the end of rehabilitation and at follow-up. The effect size was determined using Cohen’s *d .*

**Results:**

Exercise capacity and exercise time significantly decresead between end of rehabilitation and at follow-up *(p* < .0001 for both). A significant reduction in shoulder flexion repetitions (*p* = .006) was observed as well as reduced health-related quality of life in the Short Form-36 dimensions Physical Function (*p* = .042), Mental Health (*p* = .030), and Mental Component Score (*p* = .035). There were, however, no changes regarding objective and subjective physical activity measurements.

**Conclusion:**

In older patients with permanent atrial fibrillation, previously achieved improvements from physiotherapist-led exercise-based cardiac rehabilitation in physical fitness and muscle function were lost, and health-related quality of life was impaired after ending the rehabilitation period. A strategy for conserving improvements after a rehabilitation period is essential.

## Introduction

Atrial fibrillation (AF) is the most common clinically significant arrythmia in adults. AF increases with age, is more common in men, and the prevalence is > 3% in the adult population and > 20% in ages above 80 years; both incidence and prevalence is expected to increase worldwide. The proportion of permanent AF is estimated to 30% [1–3]. In older adults, AF has clinically significant negative impact on physical fitness and health-related quality of life (HR-QoL) [4–7]. Two recent meta-analyses [8, 9] conclude that cardiac rehabilitation in patients with AF can increase physical fitness and improve some aspects of HR-QoL [8]. In line with that conclusion, we recently showed in a randomized controlled trial that 3 months of physiotherapist-led exercise-based cardiac rehabilitation (PT-X) improved physical fitness in older patients with permanent AF and several co-morbidities, while physical activity on prescription did not [10].

Although PT-X improves physical fitness and muscle function in elderly patients with permanent AF and concomitant disease with considerable pharmacotherapy, little is known about the consequences when the rehabilitation period ends. In the present study, our primary aim was therefore to explore the consequences in terms of physical fitness, physical activity level, and HR-QoL among patients with permanent AF after ending a period of PT-X [10].

## Methods

We conducted a 3 months follow-up after the cessation of 3 months of PT-X in a randomized controlled study including central circulatory interval exercise and circuit training of muscle function at two 60-min hospital-based sessions, along with two home-based exercise sessions per week, as previously described in detail [10].

### Patients

All 40 eligible patients (10 women), who had completed the PT-X part of the randomized controlled study [10], were invited to participate in a follow-up study investigating the effects of 3 months without training. The patients received both written and verbal information, and provided written informed consent to participate. The investigation conformed to the declaration of Helsinki, was approved by the Regional Ethics Committee of Gothenburg, and was retrospectively registred at ClinicalTrials.gov Identifier: NCT02493400. First posted 09/07/2015. Registred in FoUvgr project 124,611. First posted 28/04/2013.

### Protocol

During the 3-month period following PT-X, the patients were asked to avoid participation in any organised exercise program that could improve physical fitness (aerobic capacity and muscular function). After this pause the patients were motivated and supported to regain their exercise habits through contact with local gym and patient associations.

### Tests

The testing procedures followed the same protocol as previously described in detail [10]. The tests were performed by physiotherapists blinded to the prior randomisation to PT-X or physical exercise on prescription. The primary outcome measure was maximum exercise capacity, assessed using a symptom-limited ergometer cycle test (Monark ergometer 839e; Monark, Varberg, Sweden) [11]. The workload began at 25 W, and was increased by 25 W every 4.5 min until the patient’s rated perceived exertion was 17 (very heavy) on the Borg scale [12]. At each workload, the patient’s heart rate and blood pressure were measured.

The secondary outcome measures included muscular endurance, physical activity level, and HR-QoL.

The muscular endurance test involved unilateral isoinertial shoulder flexion in a sitting position with a dumbbell (2 kg for women, and 3 kg for men); bilateral isometric shoulder abduction in a sitting position with a dumbbell (1 kg for both women and men); and an unilateral isoinertial heel-lift with a straight knee, on a 10° tilted wedge, with shoes on [13].

Physical activity level was measured using an accelerometer (Actigraph® GT3x+; Actigraph, Pensacola, Florida, USA). Patients were instructed to wear the accelerometer throughout the whole day for 7 days, except when taking a shower or bath [14]. Accelerometer data were calculated using the algorithm validated by Choi et al. [15]. The accelerometer measured the patient’s MET-minutes per week (MET level × minutes of activity × events per week), and one metabolic equivalent of tasks (1 MET) was equal to an oxygen uptake (VO_2_) of 3.5 mL × kg^− 1^ × min^− 1^. Physical activity was also self-reported using the Swedish version of the Short Form International Physical Activity Questionnaire (IPAQ) [16]. IPAQ categories were low (1), medium (2), and high (3) physical activity levels. For self-reported physical activity level during detraining, we also used the Saltin-Grimby’s 6-grade physical activity level scale, which has been previously validated [17].

HR-QoL was measured using the Swedish version of the Short Form-36 Health Survey Questionnaire (SF-36) [18].

### Statistics

Statistical analyses were performed using the Statistical Package for Social Science (IBM Corp. Released 2013. IBM SPSS Statistics for Windows, Version 28.0.; Armonk, NY, USA). Ratio and interval data are presented as mean (SD) and median (interquartile range), ordinal data as median (range), and nominal data as absolute and relative numbers. Data distribution was checked with Shapiro-Wilk’s test and normality was assumed if *p* > .05. Although some variables showed normal distribution, the Wilcoxon Signed Rank test was used to analyse within-group differences. The effect size was calculated using the z-value divided by the square root of N (*N* = total number of cases). A *d* value of ~ 0.2 indicated a small effect size, ~ 0.5 medium, and ~ 0.8 large [19, 20]. For changes in SF-36 dimensions, however, a 5-point difference is “clinically and socially relevant”, 10 points a “moderate”, and 20 points “a very large” one [21]. Differences were considered statistically significant when *p* was *<*.05*.*

## Results

Of the 40 eligible patients 38 (95%) followed instructions and completed follow-up (10 women), while 2 patients withdrew participation due to medical causes or unwillingness. Their mean age (SD) was 75 [4] years.

Table 1 summarizes the patients’ demographic data and clinical characteristics, and Table 2 the patient’s pharmacological therapies. Around 90% received some form of stroke prophylaxis (mainly oral anticoagulation), > 80% required heart rate regulation (mainly beta-blockers), and the majority used renin-angiotensin-inhibiting therapy.

**Table 1:** Demographic data, left ventricular function, and co-morbidities at follow-up

	PT-X (*n* = 38)
Male/female, n	28/10
Age, years	75 (4)
Weight, kg	87 (14)
Height, m	1.76 (0.08)
Body mass index, kg/m^2^	28 (4)
LV-EF, %	54 (1)
***Comorbidity***	
Myocardial infarction	1 (3%)
TIA	–
Stroke	3 (8%)
Diabetes mellitus type 2	2 (5%)
Hypertension	11 (29%)
Hyperlipidaemias	4 (10%)
COPD	1 (3%)
Asthma	2 (5%)
Gout	2 (5%)
Osteoarthritis	2 (5%)
Psoriasis	2 (5%)
Hypothyroidism	1 (3%)
Rheumatoid arthritis	1 (3%)
Spinal stenosis	1 (3%)
Osteoporosis	1 (3%)
***Surgical Procedures***	
Mitral valve replacement	1 (3%)
Aortic valve replacement	–
CABG	–

Data are presented as mean (SD) or n (%)

*Abbreviations: PT-X* Physiotherapist-led exercise-based cardiac rehabilitation, *LV-EF* Left ventricular ejection fraction, *TIA* Transient ischemic attack, *COPD* Chronic obstructive pulmonary disease, *CABG* Coronary aortic bypass grafting, *SD* Standard deviation

**Table 2:** Pharmacotherapy after the PT-X intervention period

	(*n* = 38)
Anticoagulants	35 (92%)
Platelet inhibitors	2 (5%)
Heart rate regulators ^a^	32 (84%)
Calcium channel antagonists ^b^	4 (11%)
ACE-I/ARB	24 (63%)
Alpha-receptor antagonists	–
Diuretics	9 (24%)
Nitrates	3 (8%)
Potassium supplement	2 (5%)
Oral antidiabetics	2 (5%)
Lipid modulators	17 (45%)
Thyroid hormone	3 (8%)
COPD medication	3 (8%)

Data are presented as n (%)

*Abbreviations: PT-X* Physiotherapist-led exercise-based cardiac rehabilitation, *ACEI* Angiotensin-converting enzyme inhibitor, *ARB* Angiotensin II-receptor antagonist, *COPD* Chronic obstructive pulmonary disease

^a^Beta-blockers, the calcium channel antagonists verapamil, diltiazem, and digoxin

^b^Calcium antagonists, except verapamil and diltiazem

We compared data from the tests performed immediately after the PT-X intervention period with those obtained at the 3-months follow-up.

### Primary outcome measure - physical fitness

At follow-up 3 months after the end of the PT-X period there was a significant decrease in physical fitness in terms of maximum workload (on average − 9 W) and exercise time (on average − 102 s); *p* < .0001 for both measures. The decrease in maximum workload and exercise time showed a moderate effect size (Table 3).

**Table 3:** Comparisons of physical fitness and physical activity after the intervention and at 3-months follow-up

	After intervention		At 3 months follow-up			
	PT-X		PT-X		*p* value	C’s *d*
	mean (SD)	median (interquartile range)	mean (SD)	median (interquartile range)		
Exercise Tolerance Test, primary end-point						
Maximum workload, W	103 (27)	100 (78–122)	**94 (27)**	**88 (75–112)**	**<.0001**	**0.49**
Exercise Tolerance Test, secondary end-points						
HR rest, bpm	79 (16)	79 (68–92)	77 (13)	77 (65–87)	.45	0.09
SBP rest, mmHg	139 (20)	137 (124–152)	136 (20)	132 (126–142)	.46	0.08
DBP rest, mmHg	77 (11)	78 (70–82	78 (13)	78 (68–82)	.58	0.06
Exercise time, s	1125 (297)	1086 (867–1350)	**1023 (300)**	**958 (810–1207)**	**<.0001**	**0.50**
HR at RPE 17, bpm	141 (28)	145 (126–158)	− 140 (28)	146 (115–157)	.91	0.01
Max SBP, mmHg	172 (28)	174 (154–194)	165 (24)	168 (149–182)	.10	0.19
Shoulder abduction, s	88 (44)	81 (57–104	81 (30)	78 (62–96)	.74	0.04
Shoulder flexion right, reps	35 (21)	30 (21–43)	33 (19)	30 (23–38)	.24	0.14
Shoulder flexion left, reps	33 (13)	31 (26–41)	**29 (12)**	**29 (21–36)**	**.006**	0.32
Heel lift right, reps	15 (11)	11 (7–19)	13 (9)	20 (6–16)	.27	0.14
Heel lift left, reps	14 (8)	12 (9–17)	12 (7)	11 (7–16)	.095	0.20
Physical Activity, secondary end-points						
IPAQ, Kcal	3684 (4302)	2183 (1153–4164)	3296 (2341)	2691 (1007–4366)	.49	0.083
IPAQ MET, min/week	2620(3188)	1653(839–2608)	2447 (2341)	1900 (734–2865)	.68	0.049
IPAQ Category	2 (1–3)	2 (1–2)	2 (1–3)	1 (1–2)	1.0	–
Accelerometer, Kcal	1975 (1096)	1679 (1285–2427)	2093 (1242)	1876 (1193–2520)	.30	0.12
Accelerometer MPA, min	134 (97)	91 (60–217)	149 (121)	107 (63–230)	.31	0.12
Accelerometer MVPA, min	139 (103)	91 (61–227)	153 (126)	108 (64–234)	.42	0.093
Accelerometer step counts, n	30,665 (15443)	26,516 (18356–39,775)	33,435 (19041)	27,594 (19651–41,893)	.32	0.12
Saltin-Grimby scale			3 (1)	3 (3–4)		

*Abbreviations: C’s d* Cohen’s d, *PT-X* Physiotherapist-led exercise-based cardiac rehabilitation, *bpm* Beats per minute, *SBP* Systolic blood pressure, *DBP* Diastolic blood pressure, *HR* heart rate, *Reps* Repetitions, *IPAQ* International Physical Activity Questionnaire, *Kcal* Kilocalorie, *MET* Metabolic equivalent, *MPA* Moderate physical activity, *MVPA* Moderate-to-vigorous physical activity, *n* Number of

### Secondary outcome measures

Muscle endurance in shoulder flexion in the left arm was also significantly reduced by on average − 4 reps; *p = .006* (Table 3).

Neighter self reported physical activity with the IPAQ questionnaire, nor the objective measures with accelerometer were significantly changed (Table 3).

There was a significant reduction of HR-QoL when measured using the SF-36 dimensions Physical Function (PF), on average – 3 points; *p = .042*, Mental Health (ME), on average – 4 points; *p = .030,* and the Mental Component Score (MCS), on average - 3 points *p = .035* (Table 4).

**Table 4:** Differences in health-related quality of life after the intervention and at 3-months follow-up

SF-36 dimension	After intervention		At 3-months follow-up		*p* value
	Mean (SD)	median (interquartile range)	Mean (SD)	median (interquartile range)	
PF	74 (18)	80 (65–87)	72 (17)	75 (64–85)	.042
RP	72 (38)	100 (59–100)	66 (42)	100 (25–100)	.46
BP	74 (24)	74 (61–100)	70 (25)	73 (51–100)	.31
GH	66 (17)	67 (57–77)	63 (19)	64 (50–77)	.060
VT	63 (20)	65 (50–80)	58 (18)	60 (45–70)	.052
SF	89 (19)	100 (87–100)	84 (22)	100 (62–100)	.16
RE	82 (34)	100 (67–100)	69 (41)	100 (33–100)	**.**055
MH	80 (19)	84 76–92)	76 (21)	80 (66–92)	.030
PCS	44 (9)	47 (39–51)	43 (9)	45 (38–50)	.35
MCS	51 (11)	54 (49–57)	47 (12)	52 (40–56)	.035

*Abbreviations: SF-36* Short form-36, *BP* Bodily pain, *GH* General health, *MH* Mental health, *PF* Physical function, *RE* Role emotional, *RP* Role physical, *SF* Social functioning, *VT* Vitality, *MCS* Mental Component Score, *PCS* Physical Component Score

## Discussion

The present study shows that improvements in physical fitness and muscle function gained by a 3-months PT-X period in elderly patients with permanent AF disappeared after a 3-months’ period without organised exercise. In addition, there was a reduction in HR-OoL. A strategy for conserving improvements after a rehabilitation period is thus essential.

Several studies among patients with cardiovascular diseases other than AF show that patients have difficulty continuing exercise and maintaining a sufficient physical activity level after finishing exercise-based cardiac rehabilitation [22–24]. The results of the present study both confirms the importance of developing a strategy for preserving achieved improvements and expands the knowledge of what happens when systematic circulatory training is not continued. Such information might strengthen the motivation for continued training both among patients and health care providers.

The physical fitness improvements following PT-X, 2 mL × kg^− 1^ × min^− 1^ measured as maximum work load, were comparable to those achieved in patients with chronic heart failure, in whom such improvements were associated with decreased mortality and hospital admissions during the follow-up period [25].

The period following the end of the PT-X period is compatible with the concept of detraining. Detraining is defined as the cessation or reduction of training, or a decrease in physical fitness caused by training cessation or reduction [26]. In the English literature, we have found no previous report addressing the impact of detraining in patients with permanent AF.

Previous investigations have shown that detraining causes central and peripheral alterations in athletes and recently trained healthy individuals [27–29]. These alterations are multifactorial, and include a reduced VO_2max_ due to reduced blood volume and higher heart rate response, which reduce stroke volume and affect cardiac output [29]. The peripheral alterations include reduced muscular capillarisation and oxidative enzyme activities, and a decreased arterial-venous oxygen difference and reduced oxygen delivery to the cells, which affect mitochondrial ATP production [27]. We also observed significant loss of muscular endurance, in line with previously reported results of detraining in patients with and without cardiovascular disease [30–32]. Our results corroborates those of Volaklis et al. [31] and Ratel et al. [32], which showed that detraining led to reversal of improvements of VO_2peak_ and muscular strength among patients with cardiovascular disease and in older adults [27, 28, 30–32]. Our study showed that detraining had similar effects in patients with permanent AF.

Furthermore, the period of detraining led to an impaired HR-QoL, with decreased scores on all SF-36 dimensions, and consequently a significant decrease both in physical function and mental health as well as in the mental component score. In some dimensions, the post-detraining scores were lower than the scores before PT-X. In three of these dimensions (Role Physical, Vitality, and Social Functioning), the SF-36 scores exhibited a reduction of at least 5 points, which is considered a clinically and socially relevant difference. Notably, the patients showed a 13-points deterioration in the Role Emotional dimension, which is considered a moderate change [21].

These findings indicate that both physical fitness and HR-QoL deteriorate if PT-X-induced improvements are not preserved.

Risom et al. [9] found no evidence that PT-X improved HR-QoL. This was also our finding in the first part of our study, in which the patients self-rated scores were similar to in the normative Swedish population of the same age range [10, 33]. Teixeira-Salmela et al. reported that HR-QoL increased with exercise training in the older population, and this increase persisted during detraining [30]. They proposed that patients felt better about their physical abilities and, therefore, adopted a more active lifestyle. That situation differs from the situation in our study, in which patients were asked to avoid organised exercise. Clearly, adherence to these instructions led to a reduction of HR-QoL. Our results are in line with the findings of an observational study, in which older individuals (> 65 years of age) participated in a detraining period after a period of organised exercise [34]. The participants received instructions similar to those given in the present study, and the results showed significant deterioration of all dimensions of the HR-QoL as measured by SF-36, as in our study [34]. These findings clearly confirm that after cessation of PT-X, it is crucial to support patients to maintain their physical fitness and HR-QoL.

### Methodological aspects and limitations

In this study, we investigated the consequences of not pursuing training after a period of PT-X in patients with permanent AF. Due to the high evidence of benefit from improved physical fitness this may seem ethically questionable. It is, however, well known that it is difficult to maintain good exercise habits, and the need for support in lifestyle changes is an important issue. Such a strategy also requires resources and needs to be supported by scientific evidence, which was scarce when this study was initiated in 2013. The results of the present study corroborates the need to continue exercising after a period of successful PT-X, and can thus serve to motivate patients to pursue this goal and health care authorities to allocate suitable resources.

## Conclusions

Although PT-X improves physical fitness also in older patients with AF and concomitant diseases, a subsequent period without systematic circulatory training has negative effects not only on physical fitness and muscle function but also on HR-QoL. A strategy for conserving improvements after a period of PT-X rehabilitation is essential.

## Data Availability

The dataset used and/or analysed for this current study may be available from the corresponding author upon resenable request. However, this is not applied for in the ethical application and must so be done before data sharing.

## Abbrevations

AF: Atrial fibrillation
HR-QoL: Health-Related Quality of Life
IPAQ: Short Form International Physical Activity Questionnaire
MET: Metabolic equivalent of task
PT-X: Physiotherapist-led exercise-based cardiac rehabilitation
PF: The Short Form-36 physical function dimension
RPE: Rate of perceived exertion (Borg’s scale 6–20)
SF-36: The Short Form-36 Health Survey Questionnaire
VO_2peak_: Highest oxygen uptake achieved
